# Alcohol, Cancer, Neurobiology, and Aviation Safety: Implications for Aerospace Medicine and Pilot Health

**DOI:** 10.7759/cureus.116547

**Published:** 2026-09-19

**Authors:** Ruben Vazquez

**Affiliations:** 1 Substance Abuse and Addiction Medicine, Independent Research, Orlando, USA

**Keywords:** aerospace medicine, alcohol use disorder (aud), aviation safety, cancer, cognitive functioning, cognitive neuroscience, human factors, human intervention motivation study, neurobiology of addiction, pilot mental health

## Abstract

Alcohol use in aviation has traditionally been addressed in relation to acute impairment and operational safety. However, the health implications of alcohol extend beyond intoxication and include carcinogenic risk, neurobiological changes associated with alcohol use disorders, and potential effects on cognitive functioning. This narrative review examines these issues within the occupational and aeromedical contexts of professional pilots. It also considers aviation-specific factors that may influence behavioral health and help-seeking, along with the role of the Human Intervention Motivation Study (HIMS) program in treatment, monitoring, recovery, and return to professional flying status. Examining these areas together provides a broader perspective on alcohol and pilot health than an impairment-focused approach. In aerospace medicine, this perspective supports continued attention to prevention, early identification, recovery, and long-term health, while maintaining aviation safety as the primary operational consideration.

## Introduction and background

Alcohol consumption remains one of the most prevalent and socially accepted psychoactive behaviors worldwide, despite an expanding body of evidence linking its use to significant health, cognitive, and occupational consequences. Historically, alcohol has occupied a complex position in society, functioning simultaneously as a culturally endorsed substance, social lubricant, and a recognized public health concern [1]. Within aviation medicine, discussions regarding alcohol have traditionally focused on its acute impairing effects and their direct implications for flight safety [2-5]. Real-world aviation accident data further demonstrate the operational relevance of alcohol and other potentially impairing substances affecting aviation safety. Botch and Johnson identified ethanol use among pilots involved in fatal civil aviation accidents, including cases in which ethanol concentrations exceeded the Federal Aviation Administration (FAA) regulatory limit [2]. In a subsequent analysis of 1,169 pilots fatally injured in civil aviation accidents between 2009 and 2013, Chaturvedi et al. reported that the National Transportation Safety Board (NTSB) identified ethanol and/or drugs, together with underlying medical conditions, as causes or contributing factors in 5% of the accidents examined [3].

Federal aviation regulations prohibit a person from acting or attempting to act as a crewmember of a civil aircraft within eight hours after consuming alcohol, while under the influence of alcohol, or while having an alcohol concentration of 0.04 or greater [6]. However, this impairment-focused perspective may overlook broader and more enduring alcohol-related risks that extend well beyond observable intoxication periods.

Recent advances in oncology and neuroscience challenge the traditional assumption that alcohol-related risks in aviation are confined solely to episodes of acute impairment. Emerging evidence suggests that alcohol exposure may exert cumulative effects on both long-term health and operational readiness. The International Agency for Research on Cancer (IARC) classifies alcoholic beverages as Group 1 carcinogens, reflecting sufficient evidence that alcohol consumption contributes to the development of multiple malignancies, including cancers of the oral cavity, pharynx, larynx, esophagus, liver, colorectum and female breast [7-9]. Importantly, accumulating evidence indicates that this elevated risk is not restricted exclusively to individuals with severe alcohol use disorder (AUD) or prolonged histories of heavy drinking. Even moderate alcohol consumption has been associated with measurable increases in cancer risk over time [8,9]. Concurrently, advances in addiction neuroscience have transformed contemporary understanding of AUD. Rather than conceptualizing problematic alcohol use solely through moral or behavioral frameworks, current evidence increasingly characterizes AUD as a chronic and relapsing condition involving dynamic interactions among neurobiological, psychological, social, and environmental factors [10].

Chronic alcohol exposure is associated with maladaptive neuroplastic changes involving the mesolimbic dopamine system, prefrontal cortex, hippocampus, and stress regulation pathways [10,11]. These alterations may influence reward processing, executive functioning, emotional regulation, memory, and vulnerability to relapse, several of which involve cognitive domains fundamental to safe aviation performance. These concerns are particularly important in aviation, a profession characterized by exceptionally high-performance standards, rigorous medical oversight, and an unwavering emphasis on safety.

Professional pilots routinely operate in environments that require sustained attention, cognitive flexibility, situational awareness, rapid information processing, effective decision-making, and precise psychomotor coordination [12]. Even subtle impairments in cognitive functioning, sleep quality, stress regulation, or overall health may have implications for operational performance [12,13]. Although most pilots do not develop substance-related disorders, occupational factors such as irregular schedules, circadian rhythm disruption, fatigue, prolonged time away from family, operational stress, and concerns regarding medical certification may interact with individual vulnerabilities in ways that influence coping behaviors and health outcomes of pilots [12,13].

One notable response to alcohol-related disorders in aviation is the Human Intervention Motivation Study (HIMS) program. Since its development in the 1970s, HIMS has evolved from early collaborative efforts addressing alcohol-related disorders among pilots into a structured recovery model emphasizing treatment, accountability, and return to professional aviation [14]. Through multidisciplinary collaboration involving treatment providers, aviation medical examiners (AMEs), peer monitors, employers, unions, and regulatory agencies, the program emphasizes early identification, structured treatment, long-term monitoring, and peer support. The HIMS model illustrates an approach in which recovery and aviation safety are treated as complementary, rather than mutually exclusive, objectives.

This narrative review synthesizes contemporary literature from oncology, addiction neuroscience, occupational health, and aerospace medicine to examine the broader implications of alcohol consumption on pilot health and aviation safety. Specifically, this review explores alcohol-related carcinogenicity, neurobiological mechanisms underlying AUD, cognitive and aeromedical considerations, occupational vulnerabilities unique to aviation, and the role of the HIMS model as a framework for recovery and professional reintegration into the aviation workforce.

The novelty of this review lies in the integration of carcinogenicity, neurobiological dysfunction, occupational influences, cognitive recovery, and evidence-based rehabilitation within a unified aeromedical perspective. By moving beyond the traditional impairment-focused paradigm that has historically dominated discussions of alcohol use in aviation, this review seeks to inform ongoing discussions regarding prevention, screening, intervention, recovery, and health promotion while contributing to the continued advancement of pilot health and aviation safety.

## Review

Methods

This narrative review synthesizes and integrates evidence from multiple disciplines relevant to alcohol consumption and aerospace medicine. A narrative review methodology was selected because the literature addressing alcohol-related risks in aviation encompasses substantial heterogeneity in study populations, methodologies, and outcomes across oncology, addiction neuroscience, occupational medicine, and aerospace medicine. These diverse bodies of evidence were not considered suitable for formal quantitative synthesis, which may have obscured important conceptual relationships among the examined disciplines.

Literature identification was conducted iteratively from August 2024 to July 2026, beginning with the doctoral research underlying this review and continuing during the development of the present manuscript. Targeted searches were performed using Google Scholar and electronic resources available through the Capitol Technology University Library. Representative search concepts included combinations of terms related to alcohol consumption, AUD, carcinogenicity, cancer, addiction neurobiology, cognitive functioning, pilots, aviation medicine, occupational health, fatigue, circadian disruption, and HIMS.

Peer-reviewed publications, contemporary reviews, foundational aviation texts, and relevant government and regulatory documents were considered for inclusion. Sources were selected based on their direct relevance to the objectives of the review, methodological rigor, historical significance, and contribution to understanding the implications of alcohol use for pilot health and aviation safety. No strict publication-date restriction was applied because foundational addiction models and historical HIMS literature were necessary for the review; however, contemporary evidence was prioritized when available.

Particular emphasis was placed on evidence addressing alcohol-related carcinogenicity, neurobiological mechanisms implicated in AUD, cognitive domains relevant to aviation performance, occupational and behavioral health considerations affecting aviation professionals, and recovery frameworks within safety-sensitive occupations. The resulting synthesis sought to identify areas of convergence across disciplines, distinguish established findings from proposed aeromedical implications, and identify gaps warranting additional pilot-specific investigation.

This manuscript was designed as a narrative review; therefore, no preregistered systematic review protocol, formal quantitative synthesis, or formal risk-of-bias assessment of individual studies was undertaken. This approach is subject to potential selection and interpretive bias and may not have identified every relevant publication. Nevertheless, it facilitates the integration of diverse evidence and the development of a conceptual framework to address alcohol-related health risks within the unique context of aerospace medicine.

Alcohol as a carcinogen

The relationship between alcohol consumption and cancer has become an increasingly important focus in public health and biomedical research. Although discussions regarding alcohol have historically centered on intoxication, addiction, and acute behavioral consequences, substantial evidence supports the conclusion that alcohol functions as a significant carcinogenic agent [7-9]. Contemporary findings suggest that the health implications of alcohol consumption extend beyond immediate impairment and encompass cumulative biological processes that may influence morbidity, mortality, and long-term occupational functioning.

The IARC, a specialized agency of the World Health Organization, classifies alcoholic beverages as Group 1 carcinogens, indicating sufficient evidence of carcinogenicity in humans. This designation places alcohol within the same category of established carcinogenic exposures as tobacco smoke, asbestos, and ionizing radiation. Although this classification does not imply equivalent levels of risk among these agents, it underscores the strength and consistency of the evidence linking alcohol consumption to cancer. Alcohol’s classification as a Group 1 carcinogen reflects one of the strongest levels of evidence available within cancer epidemiology [7-9].

Epidemiological investigations have identified causal associations between alcohol consumption and malignancies of the oral cavity, pharynx, larynx, esophagus, liver, colorectum, and breast [7-9]. Meta-analytic evidence consistently demonstrates dose-response relationships in which cancer risk generally increases with greater alcohol exposure [8,9]. Importantly, accumulating evidence indicates that this elevated risk is not restricted to individuals with severe AUD or prolonged history of heavy drinking. Even relatively low levels of alcohol consumption have been associated with measurable increases in cancer risk, particularly for breast cancer and cancers of the upper aerodigestive tract [8,9]. These observations challenge the longstanding assumption that low-to-moderate alcohol consumption is uniformly benign and suggest that alcohol-related health risks exist along a continuum of exposure. Evidence also suggests an association between alcohol consumption and pancreatic and stomach cancers, particularly at higher levels of alcohol exposure [8]. However, the strength and consistency of evidence for these sites remain less established than those for the seven cancers with recognized causal associations.

Several biological mechanisms have been proposed to explain alcohol-induced carcinogenesis. Among the most extensively studied mechanisms is the metabolism of ethanol to acetaldehyde, a highly reactive and genotoxic compound. Acetaldehyde can bind directly to DNA and form mutagenic DNA adducts that interfere with DNA replication and repair, contributing to genomic instability and malignant transformation, particularly in tissues of the upper aerodigestive tract [15]. In addition, alcohol metabolism generates reactive oxygen species that promote oxidative stress, lipid peroxidation, and DNA injury [7].

Alcohol-related carcinogenesis is influenced by chronic inflammatory processes and metabolic disruptions. Prolonged alcohol exposure is associated with persistent inflammation, altered immune function, impaired folate metabolism, and disruption of retinoic acid pathways involved in cellular growth and differentiation [7]. Hormonal mechanisms may also contribute to cancer development. Alcohol consumption has been associated with increased endogenous estrogen levels, which may promote estrogen-sensitive cellular proliferation and partially explain the observed relationship between alcohol consumption and breast cancer risk [16]. These mechanisms likely interact in complex and overlapping ways, rather than functioning independently.

The upper aerodigestive tract is one of the most consistently documented sites of alcohol-associated malignancies. Direct exposure of mucosal tissues to ethanol and acetaldehyde may increase susceptibility to cellular injury and carcinogenesis. Furthermore, alcohol frequently co-occurs with tobacco use, and numerous investigations have demonstrated synergistic effects between these exposures that substantially elevate cancer risk beyond the impact of either factor alone [15].

Liver cancer is another well-established consequence of chronic alcohol exposure. Progressive hepatic injury, involving steatosis, inflammation, fibrosis, and cirrhosis, creates a biological environment conducive to hepatocellular carcinoma. Similarly, increasing evidence supports the association between alcohol consumption and colorectal malignancies, potentially mediated through acetaldehyde exposure, inflammatory mechanisms, alterations in folate metabolism, and disruptions in the gut microbiome [8,9]. Collectively, the literature suggests that alcohol exerts carcinogenic effects on multiple organ systems through diverse yet interconnected biological pathways.

From an aerospace medicine perspective, these findings have implications that extend beyond traditional concerns regarding intoxication and regulatory compliance. The commercial aviation workforce is aging, and many pilots remain professionally active in later adulthood. Consequently, chronic disease prevention has become an increasingly relevant component of pilot health and aeromedical practices. Conditions associated with cancer diagnosis, treatment, and survivorship may influence medical certification, occupational functioning, quality of life, and long-term workforce participation.

Historically, discussions regarding alcohol use in aviation have focused on immediate operational safety. However, the carcinogenic properties of alcohol suggest that preventive medicine and health promotion initiatives warrant greater emphasis within pilot populations. Increased awareness of alcohol-related cancer risks may complement existing safety initiatives by encouraging informed decision-making, facilitating early intervention, and promoting healthier behavioral choices before significant medical complications develop.

Collectively, contemporary evidence supports the conclusion that alcohol functions not only as a psychoactive substance capable of producing acute impairment but also as a significant carcinogenic exposure with long-term health implications. Although individual risk varies according to dose, duration of exposure, genetic susceptibility, and environmental influences, the cumulative literature increasingly suggests that alcohol-related cancer risks deserve consideration alongside more widely recognized concerns, such as intoxication and addiction. For aerospace medicine professionals, integrating cancer prevention, health education, and risk communication into broader discussions of pilot wellness may represent an important evolution in promoting long-term occupational health and aviation safety.

Neurobiology of alcohol use disorder

Contemporary understandings of AUD have evolved substantially beyond earlier conceptualizations emphasizing moral weakness, poor judgment, or failures of willpower. Advances in neuroscience increasingly support the view that AUD is a chronic and relapsing condition involving complex interactions among neurobiological, psychological, social, and environmental factors. Although psychosocial factors remain important determinants of alcohol-related behavior, growing evidence suggests that repeated alcohol exposure produces measurable alterations in neural systems governing reward processing, executive functioning, stress regulation, learning, and behavioral control [10,17]. These neuroadaptive processes contribute to the development and maintenance of problematic alcohol use while perpetuating relapse vulnerability during recovery.

Alcohol stimulates dopamine release within mesolimbic reward pathways, reinforcing alcohol-seeking behavior and contributing to the development of addiction. Repeated activation of these circuits strengthens learned associations between alcohol use and reward, increasing the likelihood of future consumption. Chronic exposure contributes to tolerance and diminished responsiveness to naturally rewarding experiences, resulting in a state of reward deficiency in which alcohol assumes increasing motivational significance. Over time, alcohol use may shift from reward-driven behavior to compulsive consumption that becomes increasingly resistant to voluntary control [10].

Among the most influential contemporary models of addiction, Koob and Volkow’s neurocircuitry framework conceptualizes addiction as a cyclical process involving three interconnected stages: binge/intoxication, withdrawal/negative affect, and preoccupation/anticipation [10]. Repeated alcohol exposure progressively shifts motivation from positive reinforcement toward compulsive use driven increasingly by relief from negative emotional states and impaired executive control. This transition from positive to negative reinforcement represents an important neurobiological process underlying the persistence of alcohol use despite adverse consequences.

The concept of allostasis has further advanced our understanding of AUD. Unlike homeostasis, which refers to the maintenance of physiological stability, allostasis describes the establishment of new functional set points in response to stressors. Chronic alcohol exposure may recalibrate the brain's reward and stress systems, resulting in a persistent neurobiological imbalance characterized by heightened stress sensitivity, anxiety, irritability, dysphoria, and craving [10,18]. Importantly, these changes may persist long after alcohol consumption has ceased, contributing to ongoing relapse vulnerability despite sustained motivation for recovery. The transition from hedonic alcohol use to compulsive consumption reflects an allostatic process in which repeated alcohol exposure establishes pathological reward and stress set points.

Executive dysfunction is another important consequence of chronic alcohol exposure and is particularly relevant to the impaired executive-control processes associated with the preoccupation/anticipation stage of addiction [10]. The prefrontal cortex supports higher-order cognitive processes, including planning, working memory, judgment, inhibitory control, cognitive flexibility, set-shifting, and decision-making, which allow individuals to suppress maladaptive responses, shift between competing demands, update information, and adapt behavior as circumstances change. Neuroimaging investigations have demonstrated structural and functional alterations involving prefrontal cortical regions among individuals with AUD, including reductions in cortical volume and disruptions in functional connectivity [19]. Such changes may impair an individual's capacity to evaluate consequences, regulate their behavior, and override maladaptive responses. Although the degree of impairment varies substantially, deficits in executive functioning remain among the most consistently observed neurocognitive findings associated with moderate-to-severe AUD.

The hippocampus and related memory systems are similarly vulnerable to chronic alcohol exposure. These structures play essential roles in memory consolidation, contextual learning, and forming associations between environmental cues and behavioral responses. Alcohol-related hippocampal changes have been associated with reduced hippocampal volume, impaired neurogenesis, and deficits in learning and memory performance [11]. Such alterations may contribute not only to cognitive inefficiencies but also to the persistence of conditioned responses that trigger cravings and relapse. Environmental stimuli previously associated with alcohol use may retain their motivational power long after drinking has ceased, increasing susceptibility to recurrence.

Stress neurocircuitry is another critical component of contemporary addiction models. Increasing evidence suggests that chronic alcohol use disrupts the functioning of the hypothalamic-pituitary-adrenal (HPA) axis, a major physiological system involved in stress regulation. HPA axis dysregulation may contribute to heightened emotional reactivity, increased anxiety, impaired stress tolerance, and diminished resilience. Stress and addiction appear to function bidirectionally, with stress contributing to alcohol use, while alcohol-related neuroadaptations simultaneously impair the individual's ability to cope effectively with future stressors [10].

Recently, investigators have increasingly recognized the role of neuroinflammation in the pathophysiology of AUD. Neuroimmune activation involving microglial cells and inflammatory mediators has been associated with alterations in neural function and neurocognitive processes. Adams et al. [20] identified evidence across neuroimaging, cerebrospinal fluid, and postmortem investigations supporting alterations in central neuroimmune signaling among individuals with AUD and histories of heavy alcohol consumption. These findings suggest that neuroinflammatory mechanisms may represent an additional pathway relevant to the neurocognitive and clinical manifestations of AUD; however, further research is needed to clarify their functional role in cognitive impairment and addictive behaviors.

The neurobiology of craving has received substantial attention. Craving is increasingly understood as a multidimensional phenomenon involving cognitive, emotional, motivational, and physiological factors. Functional neuroimaging studies have demonstrated that alcohol-related cues may activate reward-related neural networks even after extended periods of abstinence. These findings suggest that relapse should not be viewed as a failure of motivation or character. Rather, relapse may be more accurately conceptualized as the interaction between enduring neurobiological vulnerabilities and environmental stressors, conditioned learning processes, and psychosocial influences [10].

Importantly, neuroadaptive changes associated with AUD are not necessarily permanent. Emerging evidence demonstrates that substantial recovery of brain structure and function may occur with sustained abstinence, evidence-based treatment, and ongoing recovery support [21]. Improvements have been observed in multiple domains, including executive functioning, memory performance, emotional regulation, and overall cognitive efficiency. However, recovery trajectories vary considerably among individuals, and some neurocognitive vulnerabilities may persist for months or years following the cessation of alcohol use [21].

From an aerospace medicine perspective, these findings warrant particular attention because many neural systems affected by alcohol exposure directly support the cognitive competencies required for safe aviation performance. Executive functioning, sustained attention, working memory, situational awareness, emotional self-regulation, and adaptive decision-making are foundational skills for pilots operating in complex and rapidly changing environments. Therefore, understanding the neurobiological foundations of AUD may enhance approaches to prevention, assessment, treatment, monitoring, and return-to-duty decision-making in aviation populations.

Collectively, contemporary neuroscience suggests that AUD is best understood as a multifaceted disorder involving dynamic interactions among reward circuitry, executive control systems, stress pathways, memory networks, neuroimmune processes and environmental influences [10]. Recognition of these mechanisms contributes to more sophisticated approaches to prevention and rehabilitation while simultaneously reducing the stigma associated with addiction. For aerospace medicine professionals, integrating neurobiological perspectives into discussions of pilot health provides a more comprehensive framework through which recovery, risk management, and occupational reintegration can be understood.

Cognitive and aeromedical implications

The cognitive demands associated with modern aviation require sustained attention, rapid information processing, effective decision-making, situational awareness, and precise psychomotor coordination. Professional pilots routinely operate in complex environments characterized by fluctuating workloads, dynamic operational conditions, and substantial responsibilities for passenger and crew safety. Consequently, factors influencing cognitive functioning, emotional regulation, fatigue, and overall neurological health warrant careful consideration in aerospace medicine. Although regulatory frameworks have appropriately emphasized the prevention of acute alcohol impairment, emerging evidence suggests that the neurocognitive consequences associated with alcohol use may extend beyond periods of intoxication and merit broader aeromedical attention.

One of the most consistently reported cognitive domains affected by chronic alcohol exposure is attention. Attention encompasses selective, divided, and sustained processes that enable individuals to monitor multiple streams of information simultaneously while maintaining their focus over prolonged periods. These abilities are fundamental to aviation operations, where pilots must continuously integrate aircraft system data, environmental cues, air traffic communications, and evolving operational requirements. Neuropsychological investigations have demonstrated that individuals with a history of prolonged alcohol misuse may exhibit deficits in sustained attention and reduced efficiency during tasks requiring continuous monitoring. Although the severity and persistence of these impairments vary considerably among individuals, attentional vulnerabilities have been documented even after acute intoxication has been resolved [21].

Working memory represents another cognitive domain that is critical to aviation performance. Working memory refers to the capacity to temporarily store, manipulate, and update information while performing cognitive activities. Pilots rely extensively on working memory when managing air traffic clearances, monitoring flight parameters, performing calculations, responding to abnormal situations, and integrating rapidly changing operational information. A recent systematic review and meta-analysis by Spinola et al. [22] demonstrated that acute alcohol administration adversely affects working memory performance across multiple experimental paradigms. While many individuals demonstrate meaningful cognitive recovery following sustained abstinence, subtle deficits may persist in some cases, particularly among those with longstanding or severe alcohol-related neurobiological changes.

Importantly, resolution of acute intoxication or withdrawal does not necessarily indicate complete neurocognitive recovery. Subtle deficits involving attention, working memory, executive functioning, processing speed, and cognitive flexibility may persist during abstinence, with recovery occurring at different rates across cognitive domains [21]. This distinction is particularly relevant in aviation, where comparatively small reductions in cognitive efficiency may become operationally significant during periods of high workload, rapidly changing conditions, or time-critical decision-making.

Executive functioning is arguably one of the most important cognitive domains in safety-sensitive professions. Executive functioning encompasses planning, judgment, prioritization, behavioral inhibition, cognitive flexibility, problem-solving, and risk assessment. These higher-order processes enable pilots to manage competing demands, adapt to unexpected circumstances, and make effective decisions in uncertain conditions. In aviation, cognitive flexibility and set-shifting are particularly important during rapidly evolving or abnormal situations that require pilots to redirect attention, reprioritize competing tasks, integrate new information, and modify an existing course of action under time pressure. Neurobiological evidence consistently implicates the prefrontal cortex as a critical substrate for executive control, and chronic alcohol exposure has been associated with structural and functional alterations in these regions [19]. Consequently, impairments in executive functioning have frequently been observed among individuals with active AUD and, in some cases, during the early stages of recovery.

Decision-making and risk evaluation are closely related areas of aeromedical significance. Aviation requires continuous assessment of environmental conditions, aircraft performance, weather influences, constraints and human performance factors. Decisions are often made under time pressure and with incomplete information. A systematic review and meta-analysis conducted by Ariesen et al. [23] found that adults with AUD exhibit measurable differences in risky decision-making compared to healthy controls, suggesting that disruptions in reward processing and executive control networks may influence the perception of risk and reward during periods of active alcohol use. These findings underscore the importance of understanding alcohol-related neurocognitive effects in contexts that require consistently sound judgment.

Information processing speed is another essential capability for aviation performance. Pilots must rapidly interpret sensory information, prioritize competing stimuli, and initiate appropriate responses to changing operational conditions to ensure flight safety. Evidence from the broader neuropsychological and neurobiological literature suggests that chronic alcohol exposure may adversely affect cognitive efficiency, including processes relevant to information processing speed, particularly among individuals with prolonged histories of heavy alcohol use [21]. Although the practical significance of these findings varies according to individual circumstances and recovery status, slower processing may influence performance during time-critical situations in which delayed responses can have operational consequences.

Sleep and circadian functioning are additional areas of concern. Although alcohol is sometimes perceived as a sleep aid because of its sedative properties, substantial evidence indicates that it disrupts sleep architecture and reduces restorative sleep quality. Alcohol consumption is associated with increased sleep fragmentation, alterations in rapid eye movement (REM) sleep, and reductions in overall sleep efficiency. These effects may be particularly consequential in aviation, where pilots contend with schedule irregularities, early departures, overnight operations, and repeated time zone transitions [13]. The interaction between alcohol-related sleep disruption and occupational fatigue may create a synergistic vulnerability that degrades performance more substantially than either factor alone does.

Situational awareness, a foundational construct in aviation human factors research, may also be influenced by alcohol-related neurocognitive changes. Situational awareness involves the perception of relevant environmental information, comprehension of its significance, and projection of future conditions. Effective situational awareness depends on the coordinated functioning of attention, working memory, information processing, and executive control. Although relatively few studies have directly examined these relationships among professional pilots, evidence from the broader neuropsychological literature suggests that impairments across multiple cognitive domains may influence this critical capability.

From an aeromedical perspective, the cognitive implications of alcohol use extend beyond questions of immediate impairment and encompass broader considerations of assessment, functional capacity and long-term occupational performance. Aerospace medicine practitioners are increasingly tasked with evaluating not only the presence or absence of disease but also the cognitive competencies required for safe aviation performance. This emphasis on functional assessment has contributed to the incorporation of neuropsychological evaluation within specialized programs such as the Human Intervention Motivation Study (HIMS), where cognitive testing may provide valuable information regarding attention, memory, executive functioning, and overall readiness to return to duty [24].

Importantly, the literature also provides reason for optimism. Accumulating evidence suggests that many alcohol-related cognitive deficits demonstrate partial or substantial improvement with sustained abstinence, evidence-based treatment, and ongoing recovery support [21]. Improvements have been observed in executive functioning, memory performance, emotional regulation, and attentional control. Neuroplasticity and behavioral adaptation may facilitate meaningful recovery trajectories, although outcomes vary according to factors such as the severity of alcohol exposure, age, co-occurring conditions, and treatment engagement.

These findings are particularly relevant to aviation because appropriately selected individuals who engage in comprehensive treatment and structured monitoring may successfully return to professional duties while maintaining high standards of safety and performance. This perspective aligns closely with the philosophy underlying the HIMS program and challenges the assumption that alcohol-related cognitive impairment is invariably permanent or incompatible with occupational reintegration [14].

Collectively, the evidence suggests that alcohol-related neurocognitive effects may influence several domains directly relevant to aviation performance, including attention, working memory, executive functioning, processing speed, sleep regulation, decision-making, and situational awareness. However, these effects should not be conceptualized as being static or universally permanent. Rather, cognitive functioning exists along a continuum shaped by the severity and duration of alcohol exposure, individual biological differences, treatment engagement and sustained recovery efforts [21]. For aerospace medicine professionals, integrating cognitive and aeromedical perspectives into discussions of alcohol use may support more comprehensive approaches to prevention, assessment, rehabilitation, and long-term occupational health among aviation professionals.

Occupational risk factors in aviation

The aviation profession is widely recognized as one of the most demanding and highly regulated occupational environments in modern society. Professional pilots are entrusted with substantial operational responsibilities and are expected to consistently perform at exceptionally high levels of cognitive, emotional and technical competence. Although the overwhelming majority of pilots demonstrate remarkable resilience and professionalism throughout their careers, researchers have increasingly recognized that certain characteristics of the aviation environment may influence psychological well-being and health-related behavior [12]. Understanding these occupational influences is important not because aviation professionals are inherently more vulnerable than other populations, but because the unique demands of the profession may interact with biological, psychological, and environmental factors in ways that affect long-term health outcomes.

One of the most frequently cited occupational stressors in aviation involves the magnitude of responsibility associated with flight operations. Pilots routinely make decisions that have significant implications for passengers, crew members, employers, and the traveling public. The requirement to maintain situational awareness, manage operational complexity, adapt to rapidly changing conditions, and respond effectively to unexpected events contributes to a professional culture characterized by accountability and high-performance expectations. While many pilots view these responsibilities as rewarding and integral to their professional identity, prolonged exposure to such demands may contribute to cumulative psychological strains over time.

Fatigue and circadian rhythm disruption are additional occupational challenges that have received considerable attention in aerospace medicine. Commercial flight operations frequently involve irregular schedules, early morning departures, overnight flights, extended duty periods, reserve assignments, and repeated transitions across several time zones. These operational realities may disrupt normal sleep-wake cycles and place physiological demands on pilots that differ substantially from those experienced in more traditional occupations such as office work. Chronic circadian disruption is associated with impaired sleep quality, mood disturbances, reduced psychological resilience, increased stress reactivity, and diminished overall well-being [13].

The implications of these disruptions may extend beyond fatigue. Sleep disturbances have long been associated with increased vulnerability to anxiety, depression, emotional dysregulation, and substance use in the general population. Consequently, some investigators have proposed that irregular schedules and chronic fatigue may function as indirect contributors to health-related behaviors by influencing coping capacities and stress tolerance [12,13]. Although most pilots successfully adapt to these occupational demands, understanding the interaction between circadian disruption and behavioral health remains an important area for future research.

Time away from family and social support systems represents another occupational characteristic with potential implications for psychological health [12]. Airline pilots may spend extended periods away from home because of multi-day trips, international operations, reserve assignments, training requirements, and career transitions. Such absences may complicate family relationships, parenting responsibilities, community involvement and social connectedness. Taken together, these occupational circumstances suggest that reduced access to family and social support during periods of occupational stress may increase the vulnerability among some pilots.

Career-related pressures may further influence pilots’ well-being. Aviation careers are often characterized by competitive advancement systems, recurrent training events, periodic economic instability, and the requirement to maintain stringent medical certification standards throughout one's professional life. Unlike many other occupations, pilots must repeatedly demonstrate both technical proficiency and medical fitness. Concerns regarding the potential impact of health disclosures on certification status may create additional stress when medical or behavioral health difficulties arise [25-27].

Recent evidence suggests that these concerns may significantly influence healthcare utilization among pilots. Hoffman et al. [25] reported that military aviators frequently acknowledged avoiding healthcare because of fears related to loss of flying status and concerns regarding aeromedical consequences. Similarly, Hoffman et al. [26] found that a broader sample of U.S. pilots may delay seeking medical care because of apprehension regarding potential certificate implications. More recently, Goodman et al. [27] identified multiple factors influencing healthcare-seeking behaviors and disclosure practices among U.S. Air Force pilots, highlighting the complex interplay among professional identity, confidentiality concerns, and perceptions of occupational risk. Collectively, these findings suggest that fear of professional consequences may inadvertently delay intervention until problems become more severe.

The role of occupational culture also warrants careful consideration. Aviation has historically emphasized discipline, self-reliance, competence, and professionalism in its training. These characteristics have contributed significantly to the industry's strong safety culture and remain important strengths of the profession. However, highly achievement-oriented environments may also create unintended barriers to discussing personal struggles or seeking assistance for behavioral health concerns among aviation professionals. Concerns regarding stigma, confidentiality, and perceptions of weakness have been identified in studies examining pilots’ attitudes toward mental health support.

Curreri [28] demonstrated that mental health literacy and self-stigma significantly predict commercial pilots' attitudes toward help-seeking. Pilots who perceive greater stigma surrounding behavioral health concerns may be less likely to access available support services despite recognizing the potential benefits of the intervention. These findings underscore the importance of educational initiatives designed to normalize help-seeking behaviors while preserving the professional values that characterize aviation culture.

Importantly, the relationship between occupational stress and AUD should be interpreted cautiously. Most individuals exposed to occupational stress do not develop problematic alcohol use, and stress alone is insufficient to explain the development of addiction. Contemporary AUD models emphasize the interaction between genetic predispositions, neurobiological mechanisms, psychological factors, environmental influences, and individual coping strategies [10]. Therefore, occupational stressors should not be viewed as direct causes of substance-related disorders but as contextual factors that may interact with broader biopsychosocial vulnerabilities.

Comparisons with other high-performance professions provide additional perspectives. Similar occupational themes have been documented among physicians, military personnel, law enforcement officers, air traffic controllers and first responders. Across these professions, recurring patterns involving high responsibility, irregular schedules, operational stress, and concerns regarding professional identity have been observed in the literature. These parallels suggest that many occupational factors relevant to aviation reflect broader challenges associated with safety-sensitive careers rather than risks unique to pilots.

Despite these challenges, aviation has numerous protective factors that may promote resilience and adaptive functioning. Professional pilots undergo extensive training, recurrent evaluations, medical surveillance, and continuous performance monitoring throughout their careers. Crew Resource Management principles, standardized operating procedures, strong professional identity, and peer support structures contribute to a culture that emphasizes teamwork, accountability, and safety.

Programs such as HIMS further demonstrate that early identification, evidence-based treatment, structured monitoring, and peer involvement can facilitate successful recovery and return to duty among pilots who experience substance-related difficulties [14]. The existence and sustained success of HIMS may represent a protective occupational resource, reinforcing the message that seeking help and maintaining recovery are compatible with continued professional identity and operational excellence.

Taken together, the literature suggests that occupational factors within aviation may influence health and behavioral outcomes through multiple pathways involving fatigue, circadian disruption, operational stress, social dynamics, organizational culture, healthcare avoidance, and barriers to help-seeking behaviors. These influences should not be interpreted deterministically but rather understood as part of a broader biopsychosocial framework that recognizes vulnerability and resilience. For aerospace medicine professionals, appreciating the complexities of the aviation work environment may support more effective prevention strategies, educational initiatives, early intervention efforts, and policies aimed at promoting long-term pilot well-being while maintaining the highest standards of safety.

The HIMS model and aerospace medicine considerations

The recognition of AUD as a chronic yet treatable condition has substantially influenced contemporary approaches to recovery within safety-sensitive professions. Historically, substance-related disorders in aviation have often been viewed through disciplinary or career-limiting lenses [14]. Concerns regarding public safety, regulatory compliance, and operational risk frequently result in limited opportunities for rehabilitation and return to professional duties. However, advances in addiction science and the growing recognition of the effectiveness of structured treatment and long-term recovery support have contributed to the development of more comprehensive approaches to substance-related disorders. Within aviation, one of the most influential examples of such an approach is the HIMS program.

Early discussions regarding rehabilitation-oriented approaches to alcohol-related disorders among pilots appeared before the formalization of HIMS. Weed [14] described evolving FAA policies and the Air Line Pilots Association's treatment efforts, illustrating an important philosophical shift away from exclusively punitive responses toward models emphasizing treatment, accountability, and recovery. Although these early initiatives lacked the comprehensive structural characteristics of modern HIMS, they established the foundation upon which subsequent recovery frameworks were built.

The HIMS program emerged in the 1970s through collaborative efforts involving airline management, pilot unions, addiction treatment professionals, AMEs, and federal regulators [14]. Its development reflected a growing recognition that substance use disorders could affect highly functioning aviation professionals and that appropriately treated individuals could safely return to productive careers. Rather than relying exclusively on punitive responses, the HIMS adopted a rehabilitation-oriented philosophy emphasizing early identification, treatment, accountability, monitoring, and long-term recovery support. Over the subsequent decades, HIMS evolved into one of the most widely recognized occupational recovery programs in aviation and is frequently cited as an exemplar of balancing public safety with compassionate, evidence-based care.

A distinguishing characteristic of HIMS is its multidisciplinary nature. Recovery within the program typically involves collaboration among addiction treatment providers, HIMS-trained AMEs, peer monitors, employers, pilot unions, neuropsychologists (i.e., when indicated), and regulatory authorities [24]. This integrated approach reflects the contemporary understanding that recovery from AUD is a multifaceted process requiring attention to the medical, psychological, occupational, and social dimensions of functioning. By incorporating expertise from multiple disciplines, the program seeks to support both individual recovery and aviation-safety objectives.

Comprehensive assessment and individualized treatment planning are foundational components of the HIMS process. Pilots entering the program typically undergo extensive evaluations designed to determine their diagnostic status, treatment needs, cognitive functioning, psychiatric comorbidities, and readiness for recovery [24]. Depending on individual circumstances, treatment may include residential rehabilitation, intensive outpatient programming, individual psychotherapy, group counseling, psychiatric consultation, family involvement, and participation in mutual-help organizations. Although treatment pathways vary according to clinical presentation and severity, the overarching objective remains the establishment of a stable foundation for sustained recovery before considering return-to-duty processes.

Long-term monitoring is another defining feature of the HIMS model [14]. Contemporary addiction science increasingly recognizes recovery as an ongoing process, rather than a discrete event [29]. Consistent with this perspective, the HIMS incorporates structured monitoring protocols designed to support abstinence, accountability, and early identification of potential concerns. Monitoring activities may include random alcohol and drug testing, treatment compliance verification, recovery documentation, periodic medical follow-ups, and communication among relevant stakeholders. Although monitoring requirements vary according to individual circumstances and regulatory considerations, the framework reflects the understanding that ongoing support and accountability contribute to favorable long-term outcomes.

Peer monitoring is frequently regarded as one of the most distinctive and influential elements of the HIMS program [14]. Peer monitors are typically pilots who possess specialized training and firsthand familiarity with both recovery principles and operational realities of aviation. Their roles often include providing support, encouragement, accountability, practical guidance, and assistance in navigating the complexities of recovery and professional reintegration. As peer monitors share professional experiences and occupational perspectives with participating pilots, they may be uniquely positioned to foster trust, reduce isolation, and facilitate communication. Research across multiple recovery settings consistently highlights the importance of social support, and the HIMS peer-monitoring component represents a practical application of these principles in a safety-sensitive environment.

The involvement of HIMS-trained AMEs constitutes another important strength of the program. These physicians function at the intersection of aerospace medicine, regulatory compliance and recovery management [24]. Responsibilities may include reviewing treatment progress, coordinating the required documentation, assessing medical fitness, monitoring ongoing recovery, and making recommendations regarding aeromedical certification. This specialized expertise helps ensure that return-to-duty decisions are informed by medical evidence and public safety considerations. Furthermore, the longitudinal relationships often established between pilots and HIMS AMEs may contribute to continuity of care throughout the monitoring process.

One of the most notable aspects of the HIMS model is its demonstrated capacity to facilitate successful return-to-duty outcomes among appropriately selected individuals. Although recovery trajectories vary considerably, and not all participants ultimately return to professional flight status, available reports suggest that pilots who successfully complete treatment and comply with monitoring requirements frequently achieve sustained recovery and safe occupational reintegration [14]. These outcomes are particularly noteworthy, given that aviation represents one of the most highly regulated and safety-sensitive professional environments. The ability to support meaningful recovery while maintaining rigorous safety standards has attracted the attention of clinicians, researchers, and policymakers interested in occupational approaches to addiction treatment.

Several factors likely contribute to the success of the HIMS. First, the program emphasizes early identification and intervention, thereby reducing the likelihood that difficulties remain undetected for prolonged periods. Second, its multidisciplinary structure addresses the complex biopsychosocial dimensions of addiction. Third, the integration of support and accountability mechanisms promotes both recovery engagement and public confidence. Finally, the program's emphasis on long-term monitoring aligns with contemporary conceptualizations of addiction as a chronic condition requiring ongoing management rather than brief episodes of treatment alone.

Importantly, the HIMS experience challenges the false dichotomy that public safety and rehabilitation are competing priorities. Rather, the program demonstrates that evidence-based treatment, structured accountability, and regulatory oversight can coexist within a framework that preserves both human dignity and operational safety. When supported by comprehensive systems of care and monitoring, recovery may function as a protective factor within the aviation environment.

Despite its strengths, the HIMS model has several limitations. Participation in HIMS requires substantial commitment in terms of time, effort, financial resources, and personal accountability. The recovery process may involve significant emotional and professional challenges as pilots navigate treatment, monitoring, and regulatory requirements. Additionally, because the aviation environment differs considerably from many other occupational settings, caution should be exercised when generalizing HIMS outcomes to broader populations. Continued research examining long-term outcomes, predictors of successful recovery, and opportunities for program enhancement is necessary.

From a broader occupational health perspective, the HIMS model offers valuable insights into managing substance-related disorders in safety-sensitive professions. Its emphasis on rehabilitation, multidisciplinary collaboration, structured accountability, peer involvement, and longitudinal monitoring reflects contemporary understandings of recovery and demonstrates that public safety and treatment need not be mutually exclusive goals. Although adaptation to other industries would require consideration of contextual differences, the principles underlying HIMS are relevant beyond aviation.

For aerospace medicine professionals, the HIMS experience illustrates the potential benefits of integrating medical oversight, behavioral health expertise, peer support, and regulatory collaboration within a unified recovery framework. As the scientific understanding of addiction continues to evolve, programs recognizing both the chronic nature of AUD and the possibility of meaningful recovery may become increasingly important. The experience of HIMS suggests that recovery and aviation safety are not opposing objectives. However, when supported by evidence-based treatment, structured monitoring, and collaborative oversight, recovery may serve as an essential component of long-term pilot health, occupational functioning, and public safety.

Future directions for aerospace medicine

The findings reviewed in this article point to a broader question for aerospace medicine: whether alcohol should continue to be considered primarily in terms of acute impairment or as a long-term pilot health concern. Existing operational restrictions on alcohol use are essential for flight safety, but they address only one dimension of alcohol-related risk. Evidence concerning carcinogenicity, addiction neurobiology, cognitive functioning, and occupational health supports greater attention to alcohol in preventive aerospace medicine.

One area that deserves further consideration is alcohol-related health education and screening. Current aeromedical practice appropriately emphasizes fitness for duty and the identification of conditions that may affect safety of flight. Within that framework, greater attention to patterns of alcohol use over time could provide opportunities for earlier education and intervention before an alcohol-related problem progresses to the level of a substance use disorder or produces significant health consequences. This approach should complement, rather than replace, existing regulatory and clinical assessment practices.

Neurocognitive functioning also deserves continued attention in aerospace medicine, particularly when evaluating pilots with a history of AUD. Attention, executive functioning, working memory, processing speed, and decision-making are directly relevant to the cognitive demands of flight operations. However, the evidence reviewed here does not support routine neurocognitive screening of all pilots. A more appropriate direction for future research is to determine which assessment methods are most useful for pilots with clinically indicated concerns and whether longitudinal evaluation can improve understanding of cognitive recovery and readiness for return to duty.

Occupational prevention initiatives warrant further development. The aviation environment presents unique challenges involving fatigue, circadian disruption, operational responsibility, prolonged absence from home, and concerns regarding medical certification. Expanding wellness programs that emphasize stress management, resilience building, sleep health, behavioral health literacy, and confidential support pathways may complement existing safety initiatives. Importantly, efforts to reduce the stigma surrounding help-seeking behaviors should remain a priority, as concerns regarding professional consequences continue to represent barriers to early intervention [25-28].

The HIMS program offers a useful framework for future innovations. Its emphasis on multidisciplinary collaboration, evidence-based treatment, structured monitoring, peer involvement, and regulatory partnerships demonstrates that public safety and rehabilitation can coexist successfully within highly safety-sensitive environments. Lessons derived from HIMS may inform future approaches to behavioral health management in aviation and potentially provide guidance for other professions seeking to balance accountability with opportunities for recovery and reintegration into the workforce. Continued evaluation of HIMS outcomes may further identify factors associated with a successful return to duty and opportunities to optimize existing monitoring practices.

Emerging precision medicine approaches may eventually permit individualized risk stratification by integrating genetic susceptibility, cumulative alcohol exposure, neurocognitive trajectories, and occupational variables. Such innovations may enhance preventive aerospace medicine practices by facilitating the earlier identification of risk while minimizing unnecessary restrictions among pilots who demonstrate resilience and recovery. Although these approaches remain largely investigational, they represent a promising avenue for future research and clinical applications.

As a narrative review, this manuscript did not employ a preregistered systematic review protocol, formal quantitative synthesis, or individual study risk of bias assessment of individual studies. Consequently, selection bias, interpretive bias, and incomplete retrieval of relevant literature cannot be entirely excluded, and the relative weighting of individual sources may reflect the interdisciplinary objectives of this review. Additionally, much of the literature concerning alcohol-related neurobiological dysfunction originates from general population samples rather than aviation professionals, limiting the direct generalizability to pilot populations. Likewise, although evidence supporting alcohol-related carcinogenesis is robust, few investigations have examined these relationships specifically among pilots, leaving unanswered questions regarding the potential influence of aviation-specific exposures, such as circadian disruption, fatigue, and occupational stress.

Future research should prioritize longitudinal studies involving aviation populations. Prospective cohort studies examining cumulative alcohol exposure, cancer incidence, cognitive trajectories, treatment engagement, and occupational outcomes would substantially strengthen the evidence base for aeromedical decision-making. Additional studies examining the interactions between alcohol-related sleep disruption, fatigue, and operational performance are similarly warranted. Longitudinal studies of pilots in recovery incorporating comprehensive neuropsychological assessment may further clarify the trajectory of cognitive recovery and determine whether findings derived largely from general AUD populations translate directly to the unique cognitive and operational demands of professional aviation. Neuroimaging investigations involving pilots with varying alcohol exposure histories may further clarify the extent to which the observed cognitive changes reflect alcohol-related mechanisms versus broader occupational influences. Research evaluating behavioral health interventions and educational initiatives specific to aviation professionals also represents an important area for development. Understanding whether enhanced communication regarding the carcinogenic and neurobiological effects of alcohol influences health behaviors may inform future prevention strategies. Likewise, continued examination of recovery trajectories among pilots participating in programs such as HIMS may help refine monitoring practices and identify factors associated with sustained recovery and successful occupational reintegration into aviation.

Ultimately, the future of aerospace medicine may require continued evolution from models focused primarily on the identification of disease and acute impairment to approaches emphasizing longitudinal wellness, resilience, prevention, and functional recovery. Such evolution does not diminish the importance of operational safety; rather, it broadens the scope of factors considered essential for maintaining it. By recognizing that pilot health and public safety are deeply interconnected, aerospace medicine has the opportunity to adopt more comprehensive and compassionate approaches that promote both individual well-being and system-wide safety outcomes.

## Conclusions

Alcohol use in aviation has traditionally been viewed primarily through the lens of acute impairment and its potential effects on flight operations. The evidence reviewed in this article demonstrates that this is a broader issue. Alcohol consumption is associated with long-term health concerns involving carcinogenic risk, changes in neurobiological functioning, and cognitive effects that may be particularly relevant in a profession where sustained attention, judgment, and decision-making are essential.

For aerospace medicine, these findings support looking beyond immediate impairment when considering alcohol consumption and pilot health. Prevention, education, early identification, treatment, and recovery all play a role in protecting pilots’ long-term health. The experience of HIMS also demonstrates that pilots with AUD can receive treatment, participate in structured monitoring, and successfully return to professional flying when the appropriate aeromedical standards are met. Continued research involving aviation populations is important for determining how alcohol-related health risks and recovery can be better understood within the unique demands of the profession. Ultimately, protecting pilot health and maintaining aviation safety should remain complementary objectives of the aviation industry.
